# Comparative analysis of 70 SMILE and 70 toric ICL eyes for myopic oblique astigmatism: a matched cohort study from an initial pool of 11,929 eyes

**DOI:** 10.1007/s10792-026-03986-z

**Published:** 2026-02-16

**Authors:** Abdelrahman Assaf, Baha A. Alsaify, Leonie Troeber, Amir Javadi, Rainer Wiltfang, Martin Bechmann, Klio Becker, Nikolaus Feucht

**Affiliations:** 1 Smile Eyes Eye Clinic, Munich Airport Center, Munich, Germany; 2 Smile Eyes Eye Clinic, Trier, Germany; 3 Department of Ophthalmology, University Hospital Ulm, Ulm University, Prittwitzstraße 43, 89075 Ulm, Germany; 4 Department of Ophthalmology, University Hospital Rechts Der Isar, Technical University of Munich, Munich, Germany; 5 Network Engineering and Security Department, Jordan University of Science and Technology, Irbid, 22110 Jordan; 6 Mathematics and Computer Science Department, University of Puget Sound, Tacoma, WA 98416 USA

**Keywords:** Small incision lenticule extraction, SMILE, EVO + ICL^TM^, Oblique astigmatism, Phakic intraocular lenses(pIOLs)

## Abstract

**Purpose:**

To compare visual, refractive, and vector-based astigmatic outcomes between Small Incision Lenticule Extraction (SMILE) and toric Implantable Collamer Lens (ICL) implantation in patients with myopic oblique astigmatism.

**Methods:**

In this retrospective, matched cohort study, 140 eyes (70 SMILE, 70 toric ICL) with oblique astigmatism were treated at a single center. Groups were matched preoperatively for refractive cylinder and spherical equivalent within ± 0.50 D. Postoperative outcomes at six weeks were assessed, including uncorrected and corrected distance visual acuity (UDVA, CDVA), spherical equivalent (SEQ), astigmatism correction using vector analysis (Alpins method), and safety and efficacy indices. A post hoc power analysis was performed for the astigmatic correction index.

**Results:**

The efficacy index was 0.97 ± 0.17 in the ICL group and 0.94 ± 0.15 in the SMILE group. The safety index was 1.02 ± 0.11 (ICL) versus 1.01 ± 0.09 (SMILE). UDVA equal to or better than preoperative CDVA was achieved in 83% of ICL eyes and 79% of SMILE eyes. SEQ within ± 0.50 D was observed in 85.7% (ICL) versus 81.4% (SMILE). The astigmatic correction index was 0.97 for ICL and 1.04 for SMILE. Linear regression showed stronger correlation between target and achieved astigmatism in the ICL group (slope = 1.04, R^2^ = 0.77) compared to SMILE (slope = 0.76, R^2^ = 0.67). Power analysis confirmed 80.5% power to detect clinically meaningful differences.

**Conclusions:**

Both SMILE and toric ICL are effective and safe for correcting myopic oblique astigmatism. However, toric ICL demonstrated slightly greater precision in axis alignment and refractive predictability, supporting its use in cases of high oblique astigmatism where rotational accuracy is critical.

## Introduction

Myopia is one of the most common refractive error worldwide, affecting billions of individuals across all age groups. Although spectacles and contact lenses remain the primary non-surgical management strategies, corneal laser refractive procedures have become well-established alternatives. In recent years, Small Incision Lenticule Extraction (SMILE)—a type of Keratorefractive Lenticule Extraction (KLEx) has emerged as a minimally invasive alternative [1, 2]. SMILE employs a femtosecond laser to create a stromal lenticule, which is extracted through a small peripheral incision. This technique offers several potential advantages over conventional methods, including better preservation of corneal biomechanics and a lower incidence of postoperative dry eye [3].

However, not all patients are suitable candidates for corneal refractive surgery. Factors such as high myopia, thin corneas, or abnormal corneal topography may contraindicate laser-based approaches. Additionally, preservation of the native corneal architecture is clinically relevant, as procedures such as SMILE can limit the suitability for subsequent intraocular interventions, including the implantation of multifocal or extended depth-of-focus (EDOF) intraocular lenses. For such cases, phakic intraocular lenses (pIOLs)—and in our centers the EVO + ICL™ Implantable Collamer Lens™ (Staar Surgical®)—offer a viable and effective alternative. The EVO + ICL™ is CE-certified and has received regulatory approval from the EMA and FDA since 1997, with over two million lenses implanted worldwide.

In clinical practice, correction of oblique astigmatism appears to be associated with a higher rate of retreatments (data on file), a finding echoed by limited existing literature. Oblique astigmatism poses specific challenges due to its increased sensitivity to axis misalignment and the relative paucity of comparative outcome data for SMILE and toric ICL in this subgroup. Establishing the relative efficacy and predictability of these techniques is therefore essential for informed clinical decision-making.

Oblique astigmatism, typically defined as axes between 31°–59° and 121°–149°, accounts for approximately 10–20% of all astigmatic presentations [4, 5]. Although often grouped with other astigmatic types, its optical characteristics are distinct. It is generally considered separately from with-the-rule (WTR) and against-the-rule (ATR) patterns, which are defined based on their orientation relative to the vertical meridian [6].

To better understand clinical outcomes in this subset of patients, we conducted a retrospective analysis at Smile Eyes Augenärzte Airport, Munich Airport, evaluating patients treated between January 1, 2018, and October 31, 2023. During this period, 3,679 eyes received toric ICL implantation and 8,250 eyes underwent the SMILE procedure for the correction of myopia or hyperopia, with or without astigmatism. The present study focuses specifically on the subgroup of eyes with oblique astigmatism treated by either modality.

## Study design and population methods

The study was conducted in accordance with the tenets of the Declaration of Helsinki and received approval from the Ethics Committee of the Bavarian Medical Association. The SMILE cohort was approved under the reference number EK-25035-01, and the ICL cohort was approved under the reference number EK-25011-01. All procedures adhered to the ethical standards set forth in the Declaration of Helsinki.

We compared the postoperative refractive outcomes of patients undergoing either Small Incision Lenticule Extraction (SMILE) or EVO + ICL™ implantation for the correction of myopia with either regular (orthogonal) or oblique astigmatism. All SMILE procedures were performed using the VisuMax 800 femtosecond laser system (Carl Zeiss Meditec AG®), while all ICL implantations utilized the EVO + ICL™ model (Staar Surgical®). Axis alignment and cyclotorsion control differed between groups, with combined manual and digital axis marking applied in the ICL group, whereas SMILE treatments relied on visual-axis–based centration using the Chang–Waring chord without additional corneal axis marking.

A total of 8,250 eyes were included in the SMILE group (59% female; n = 3,449), and 3,679 eyes in the ICL group (67% female; n = 1,666). The mean age was 29.7 ± 4.3 years in the SMILE group and 29.3 ± 4.5 years in the ICL group.

### Matching and group division

To enhance comparability, preoperative spherical and cylindrical refractive values were matched between groups within a tolerance of ± 0.50 diopters. Patients were then subdivided into two astigmatism categories based on preoperative refractive axis:

- *Regular astigmatism group* Axis between 90° ± 30° or 180° ± 30°
- *Oblique astigmatism group* Axis outside these ranges (i.e., 31°–59° and 121°–149°)

Patients were excluded if they:

- Lacked postoperative refractive measurements at ≥ 6 weeks
- Were older than 38 years at the time of surgery to minimize the confounding effects of early presbyopia, which may lead to non-plano refractive targets (e.g., monovision strategies) and limit comparability of postoperative visual outcomes.

### Preoperative characteristics

The maximum preoperative spherical error was − 18.00 D in the ICL group and − 9.5 D in the SMILE group. The maximum cylindrical error was − 7.70 D (ICL) and − 4.20 D (SMILE).

### Statistical analysis

All analyses were performed using Microsoft Excel (Microsoft Corp., Redmond, WA) and IBM SPSS Statistics (Version 29.0.2.0(20), IBM Corp., Armonk, NY). IBM SPSS Statistics for Windows, Version 29.0.2.0. Normality of data distribution was assessed using standard tests. For non-normally distributed variables, data are presented as median (interquartile range) and compared using the Mann–Whitney U test (between groups) or Wilcoxon signed-rank test (within-group comparisons). For normally distributed variables, mean ± standard deviation is reported, and comparisons were made using Student’s t-test. A *p*-value < 0.05 was considered statistically significant. Diagrams and visual representations of data were generated using ASSORT® and mEYEstro [7].

Vector analysis was performed using the Wilcox–Holladay–Wang–Koch (WHWK) Statistical Software for R Project, as recommended by the ASCRS/ESCRS standards for astigmatic analysis [8, 9].

The software automatically generates double-angle convex polygon plots that display the centroid (refractive geometric mean) and 95% confidence boundaries for the astigmatic correction vectors.

## Results

A total of 70 eyes from 70 patients per group with oblique astigmatism were analyzed. The primary hypothesis was that refractive precision and axis stability differ between toric ICL and SMILE in the correction of myopic oblique astigmatism.

Preoperative spherical equivalent (SEA) was comparable between SMILE (− 5.10 ± 1.16 D) and ICL (− 5.35 ± 1.18 D; *p* = 0.2, Mann–Whitney U test).

Postoperative spherical equivalent deviation (SED) at 6 weeks revealed slightly more precise emmetropization in the ICL group (0.07 ± 0.44 D) compared to SMILE (− 0.16 ± 0.41 D; *p* < 0.001, Mann–Whitney U test). Although statistically significant, this difference represented a small clinical effect (Cohen’s d = 0.21, 95% CI [− 0.56, 0.10]).

Mean cylindrical reduction was similar in both groups, with residual postoperative cylinder values of − 0.44 ± 0.35 D in the ICL group and − 0.38 ± 0.29 D in the SMILE group (*p* = 0.2, Mann–Whitney U test).

The uncorrected distance visual acuity (UDVA) (decimal) at 6 weeks was comparable between the two groups. Mean UDVA was 1.05 ± 0.209 in the ICL group and 1.00 ± 0.216 in the SMILE group (*p* = 0.178), indicating no statistically significant difference. Although the ICL group showed a slightly higher mean visual acuity, the difference was not clinically or statistically significant (Cohen’s d = − 0.21, 95% CI [− 0.56, 0.10]). Confirming no clinically relevant difference in uncorrected vision.

**Fig. 1:**
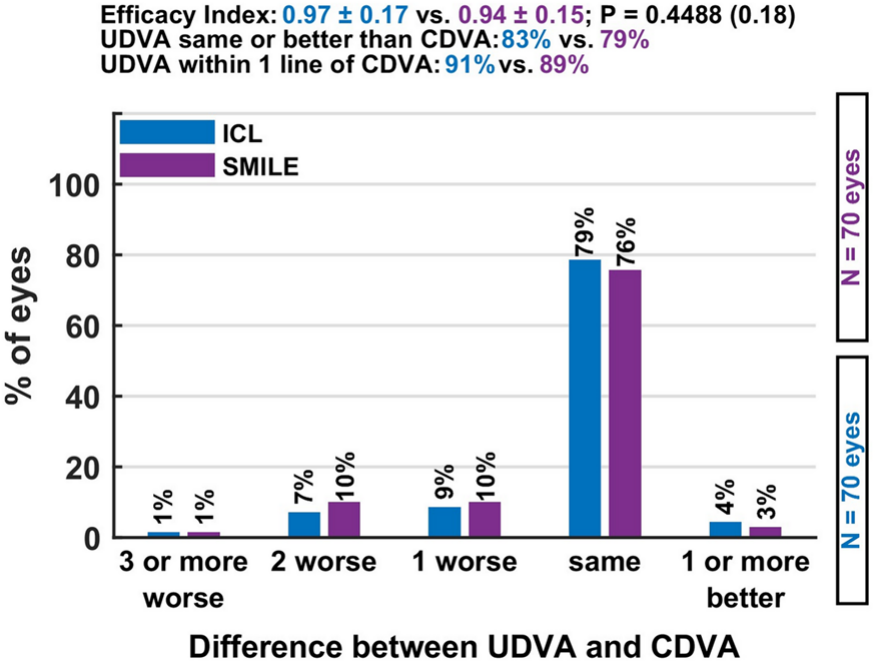
This figure illustrates the distribution of uncorrected versus preoperative corrected visual acuity, showing that most eyes achieved postoperative vision equal to or better than their preoperative best-corrected level in both groups

**Fig. 2:**
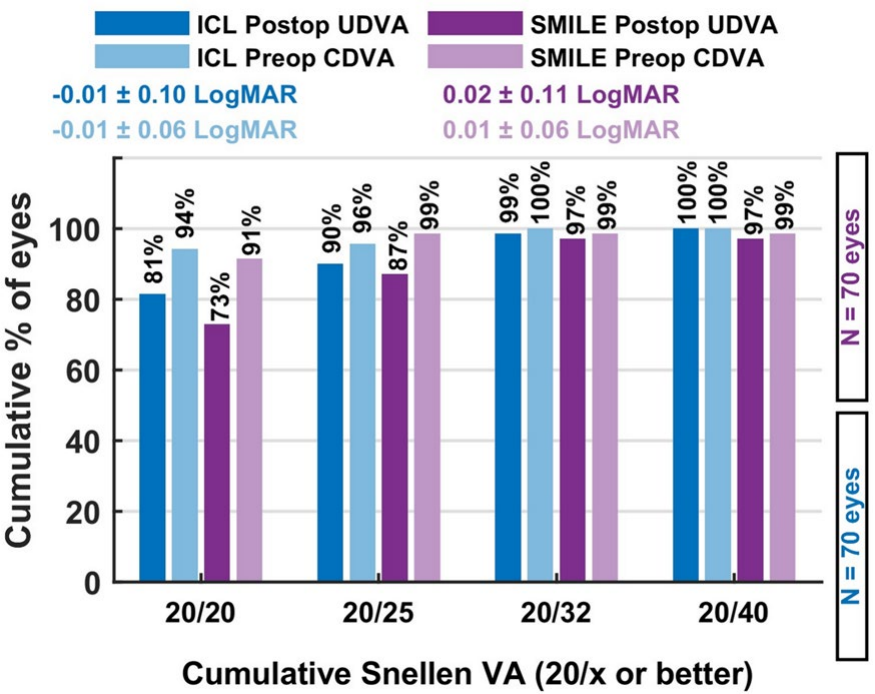
Cumulative UDVA: Percentage of eyes achieving cumulative uncorrected distance visual acuity (UDVA) of 20/20–20/40 or better, comparing preoperative CDVA and postoperative UDVA for both groups

*Efficacy* At six weeks postoperatively, both groups demonstrated excellent visual outcomes. See Fig. 1 The mean efficacy index was 0.97 ± 0.17 in the ICL group and 0.94 ± 0.15 in the SMILE group, with no statistically significant difference (*p* = 0.18). A postoperative uncorrected distance visual acuity (UDVA) equal to or better than the preoperative corrected distance visual acuity (CDVA) was achieved in 83% of ICL-treated eyes and 79% of SMILE-treated eyes. These findings confirm the strong efficacy of both procedures for correcting myopia with oblique astigmatism and indicate that each method reliably delivers high-quality functional vision within a short postoperative interval Fig. 2.

**Fig. 3:**
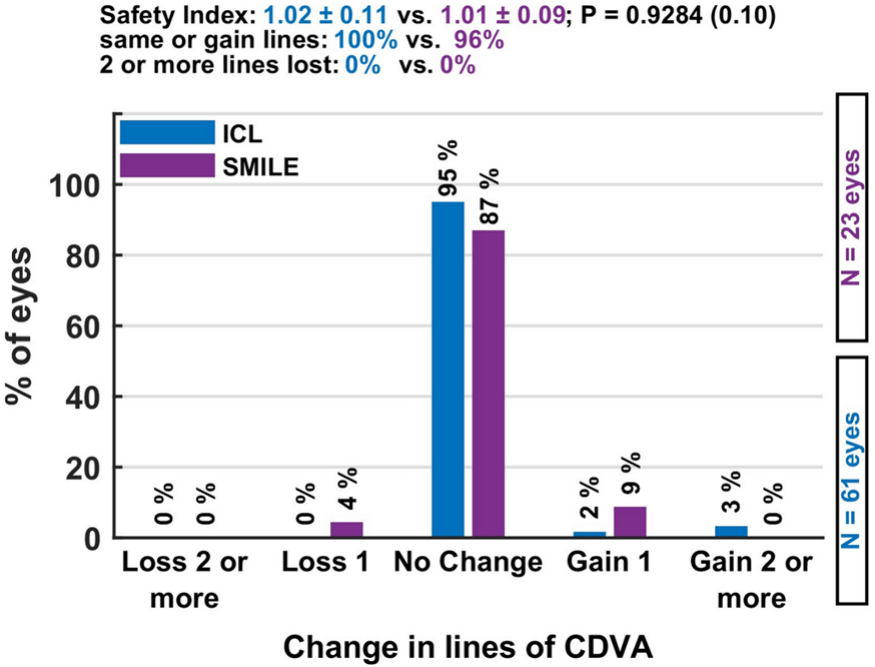
The safety graph shows that vision loss was rare in both procedures, with only minimal proportions losing one line, supporting the high safety profile of SMILE and ICL

*Safety* The safety index was 1.02 ± 0.11 in the ICL group and 1.01 ± 0.09 in the SMILE group, indicating a high level of safety in both cohorts. Notably, no eye in either group lost more than one line of CDVA, and several eyes gained lines. These results suggest that both ICL and SMILE are safe options for patients with oblique astigmatism, with minimal risk of vision loss or postoperative complications Fig. 3.

**Fig. 4:**
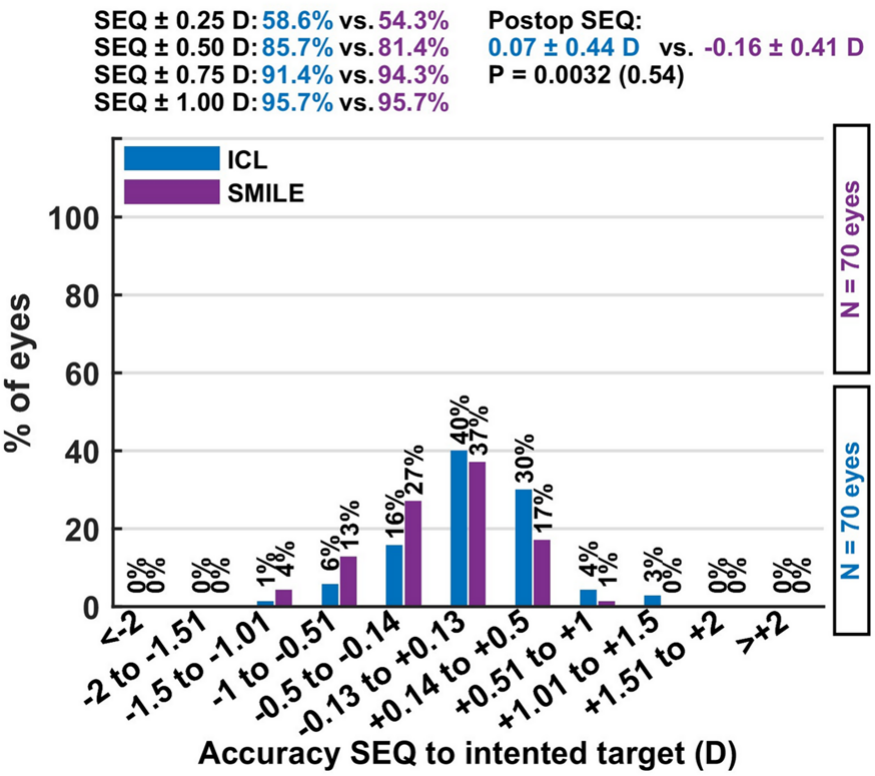
The safety graph shows that vision loss was rare in both procedures, with only minimal proportions losing one line, supporting the high safety profile of SMILE and ICL

**Fig. 5:**
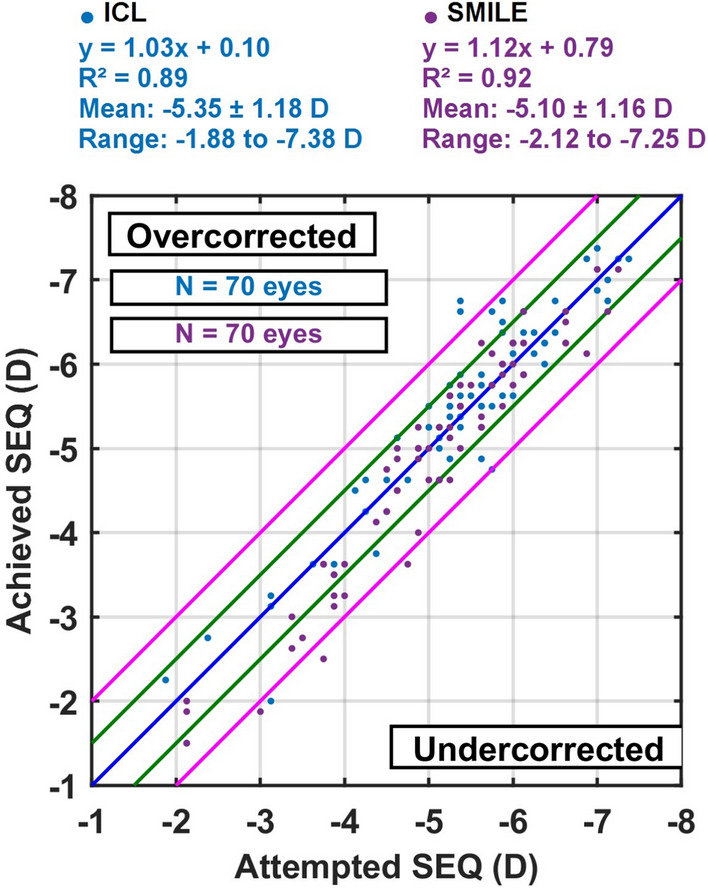
The regression plot of attempted versus achieved spherical equivalent demonstrates strong linear correlation in both groups, confirming predictable refractive performance

*Accuracy* In terms of refractive precision, 85.7% of eyes in the ICL group and 81.4% in the SMILE group achieved a postoperative spherical equivalent (SEQ) within ± 0.50 D of the target. The mean achieved SEQ was + 0.07 ± 0.44 D in the ICL group and − 0.16 ± 0.41 D in the SMILE group. These data indicate a high degree of predictability, with the SMILE group showing a slight trend toward mild undercorrection. Nevertheless, both modalities achieved excellent alignment with the intended refractive outcome, reinforcing their reliability in clinical practice Figs. 4 and 5.

### Astigmatic correction and vector analysis

Vector analysis revealed a mean correction index of 0.97 ± 0.32 in the ICL group and 1.04 ± 0.34 in the SMILE group, indicating comparable accuracy of astigmatic correction between the two procedures. The mean angle of error was 2.9° ± 18.2° for the ICL group and 3.2° ± 11.1° for the SMILE group, suggesting minimal axis deviation in both procedures, with ICL showing slightly better alignment but greater variability among eyes.

**Fig. 6:**
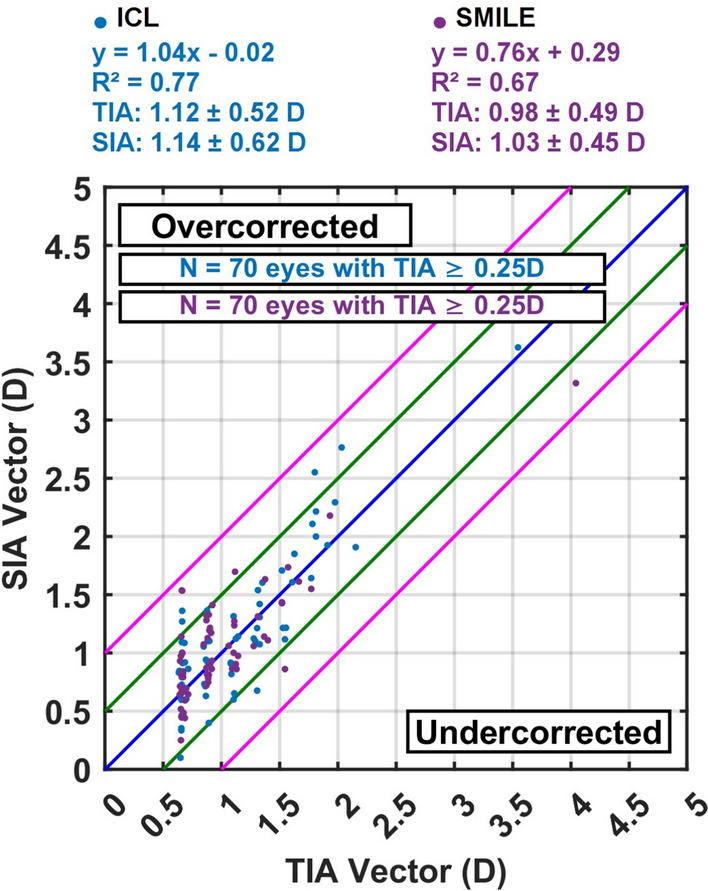
The defocus equivalent distribution confirms similar overall refractive accuracy for both techniques, with most eyes achieving values below 0.75 D, indicating low combined spherical and cylindrical residual error

The refractive surgically induced astigmatism (SIA) showed a strong correlation with the target-induced astigmatism (TIA) in both treatment groups. However, the ICL group demonstrated values that were closer to the ideal 1:1 ratio, reflecting slightly greater precision in achieving the intended astigmatic correction (Fig. 6).

**Fig. 7:**
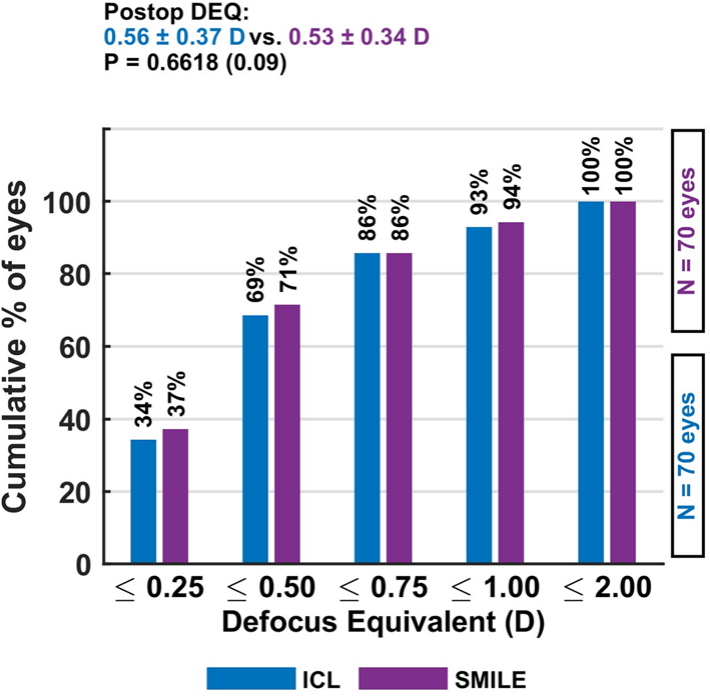
The defocus equivalent distribution confirms similar overall refractive accuracy for both techniques, with most eyes achieving values below 0.75 D, indicating low combined spherical and cylindrical residual error

The mean defocus equivalent—a combined measure of residual spherical and cylindrical refractive error—was 0.56 ± 0.37 D in the ICL group and 0.53 ± 0.34 D in the SMILE group, indicating similar levels of overall refractive accuracy. Postoperative refractive astigmatism of ≤ 0.75 D was observed in 91.4% of ICL-treated eyes and 94.3% of SMILE-treated eyes, underscoring the high effectiveness of both techniques in reducing astigmatism to clinically acceptable levels (Fig. 7).

**Fig. 8:**
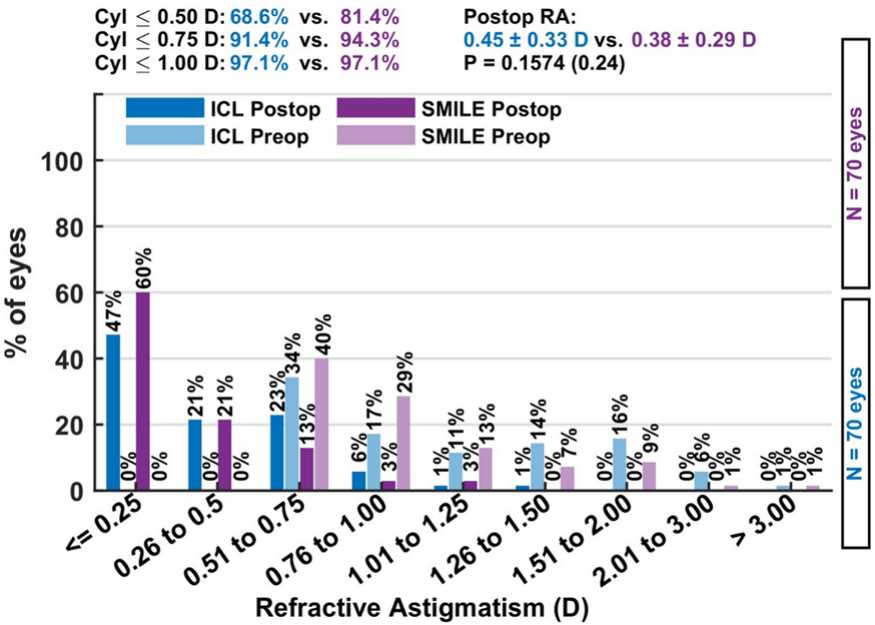
This plot presents the distribution of postoperative refractive cylinder, showing that both procedures achieved minimal residual astigmatism, with a slightly higher proportion of ICL eyes reaching ≤ 0.75 D

The mean defocus equivalent was 0.56 ± 0.37 D (ICL) and 0.53 ± 0.34 D (SMILE). Postoperative refractive astigmatism of ≤ 0.75 D was observed in 91.4% of ICL eyes and 94.3% of SMILE eyes Fig. 8.

### Astigmatic vector analysis (Alpins Method)

Vector analysis was conducted using the Alpins method to evaluate the accuracy, consistency, and axis alignment of astigmatic correction in eyes treated with either SMILE or toric ICL for myopia with oblique astigmatism.

### Surgically and target-induced astigmatism

The mean target-induced astigmatism (TIA) was 1.12 D in the ICL group and 0.98 D in the SMILE group. Corresponding surgically induced astigmatism (SIA) was 1.14 D (ICL) and 1.03 D (SMILE). Vector means and polar plots confirmed consistent magnitude alignment in both techniques. However, the regression analysis of SIA versus TIA demonstrated stronger agreement in the ICL group (slope = 1.04, R^2^ = 0.77) compared to the SMILE group (slope = 0.76, R^2^ = 0.67), indicating a systematic trend toward undercorrection in SMILE-treated eyes.

### Correction index and difference vector

**Fig. 9:**
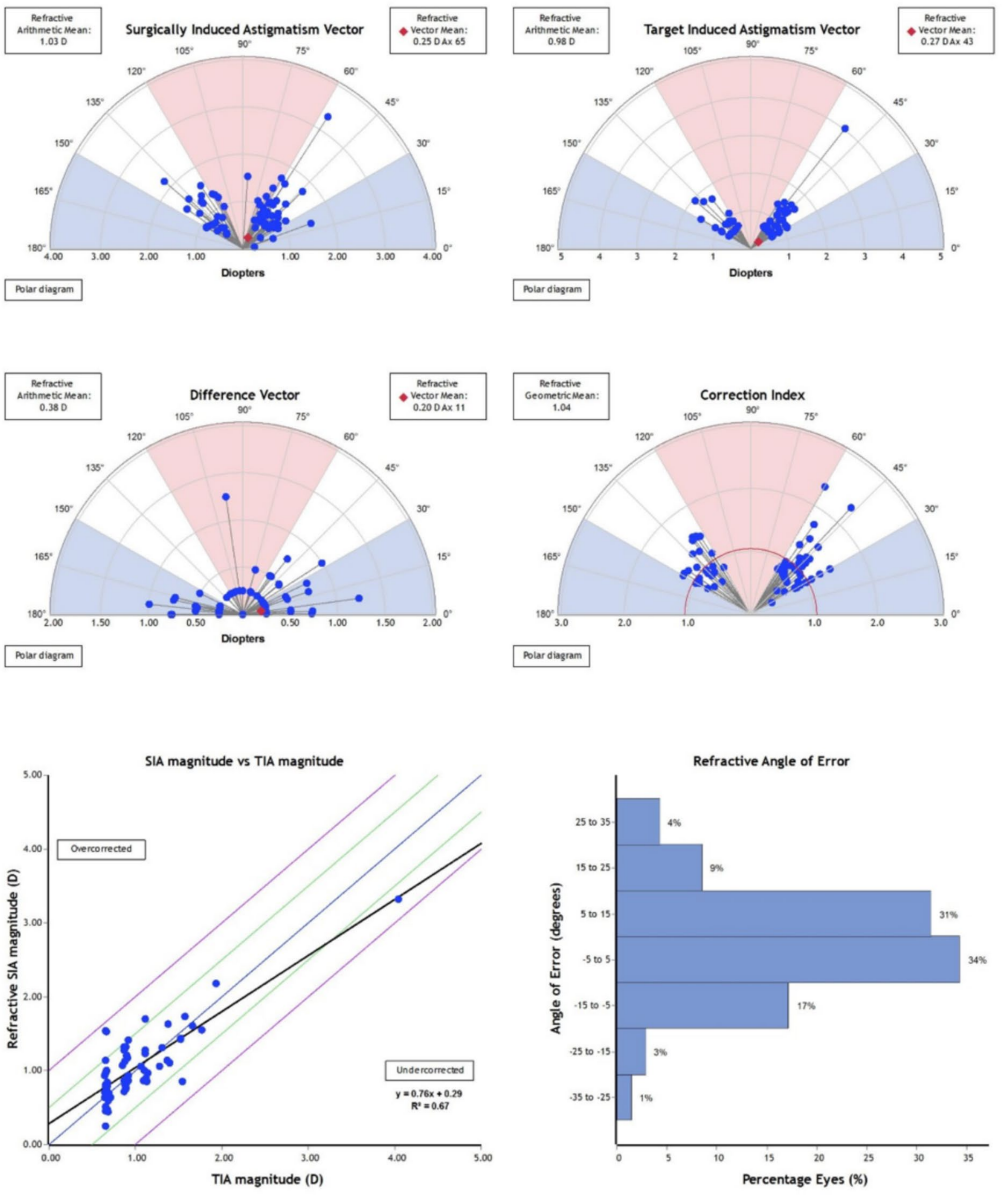
Vector analysis of astigmatic correction in eyes treated with Small Incision Lenticule Extraction (SMILE). The upper panels illustrate the Surgically Induced Astigmatism (SIA) and Target-Induced Astigmatism (TIA) vectors, while the lower panels show the Difference Vector (DV), Correction Index (CI), and the relationship between TIA and SIA magnitudes, as well as the distribution of the Angle of Error (AE).The regression plot demonstrates a slope of 0.76 (R^2^ = 0.67), indicating a mild overall tendency toward undercorrection. Notably, in eyes with astigmatism greater than 1.5 D, the SIA values tended to deviate further from the 1:1 line, suggesting reduced correction efficiency at higher cylinder powers. The AE histogram shows that 82% of eyes were within ± 15°, confirming good axis alignment overall

**Fig. 10:**
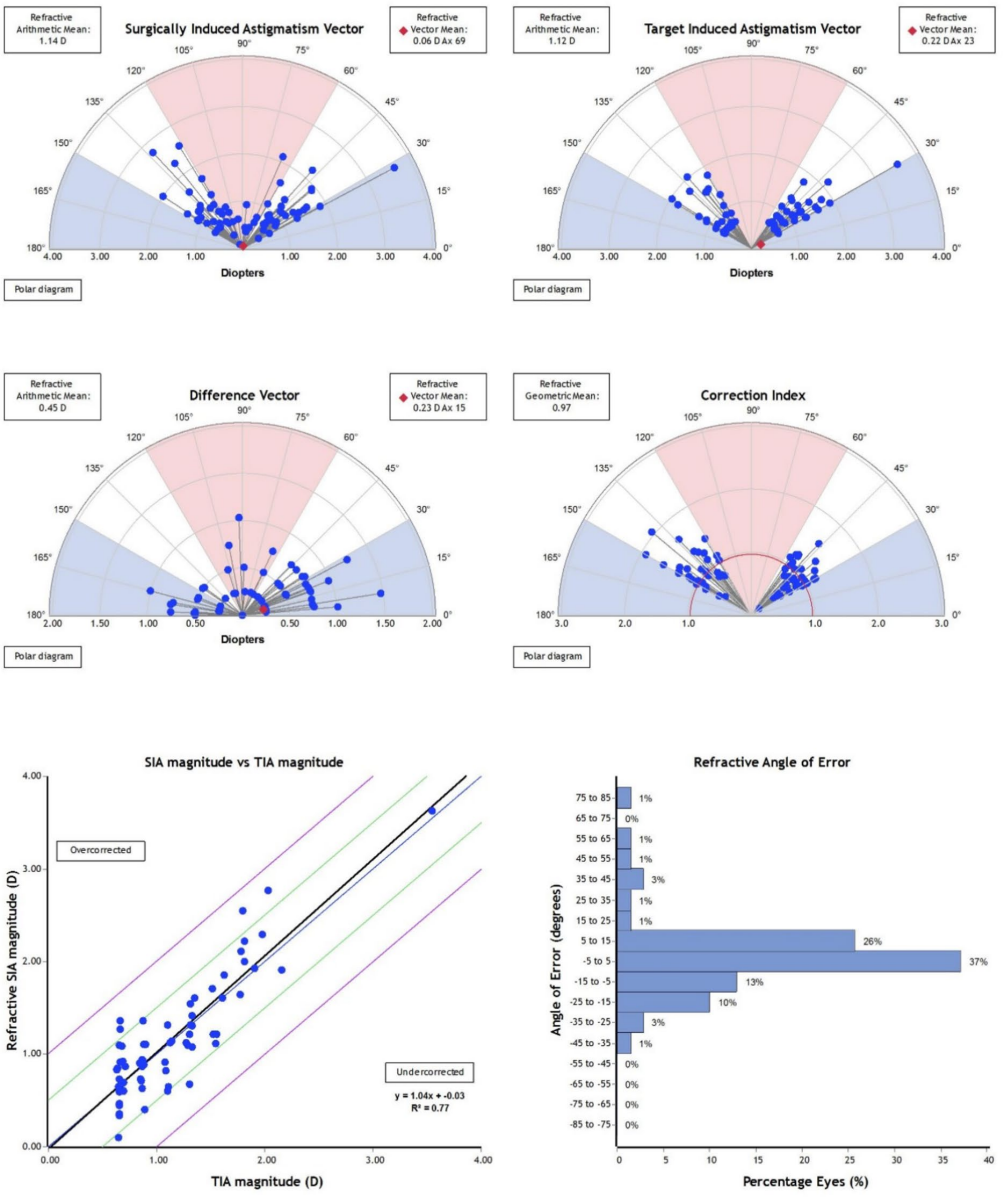
Vector analysis of astigmatic correction in eyes treated with toric implantable collamer lens (ICL) implantation.The upper panels show the Surgically Induced Astigmatism (SIA) and Target-Induced Astigmatism (TIA) vectors.The lower panel displays the Difference Vector (DV) and Correction Index (CI), while the bottom row presents the relationship between TIA and SIA magnitudes (demonstrating a slope near 1.0, R^2^ = 0.77) and the distribution of the Angle of Error (AE). Most data points cluster tightly around the 1:1 line, indicating high accuracy of the astigmatic correction and minimal axis misalignment in the ICL group

The mean correction index (CI) of 0.97 ± 0.32 in the ICL group and 1.04 ± 0.34 in the SMILE group (P = 0.48). Both groups demonstrated low difference vectors (DV), with a vector mean of 0.22 D @ 16° for ICL and 0.20 D @ 11° for SMILE. The arithmetic mean of DV was 0.45 D in ICL and 0.38 D in SMILE, indicating residual astigmatism was generally minimal in both modalities Figs. 9 and 10.

### Angle of error

The refractive angle of error (AE) was comparable between groups, with 82% of SMILE-treated eyes and 76% of ICL-treated eyes falling within ± 15° of the intended axis (*P* = 0.43). Although both procedures demonstrated acceptable alignment accuracy, SMILE-treated eyes showed a slightly higher proportion within the ideal AE range. A small number of eyes in each group exhibited AE > 15°.

### Overall performance

**Fig. 11:**
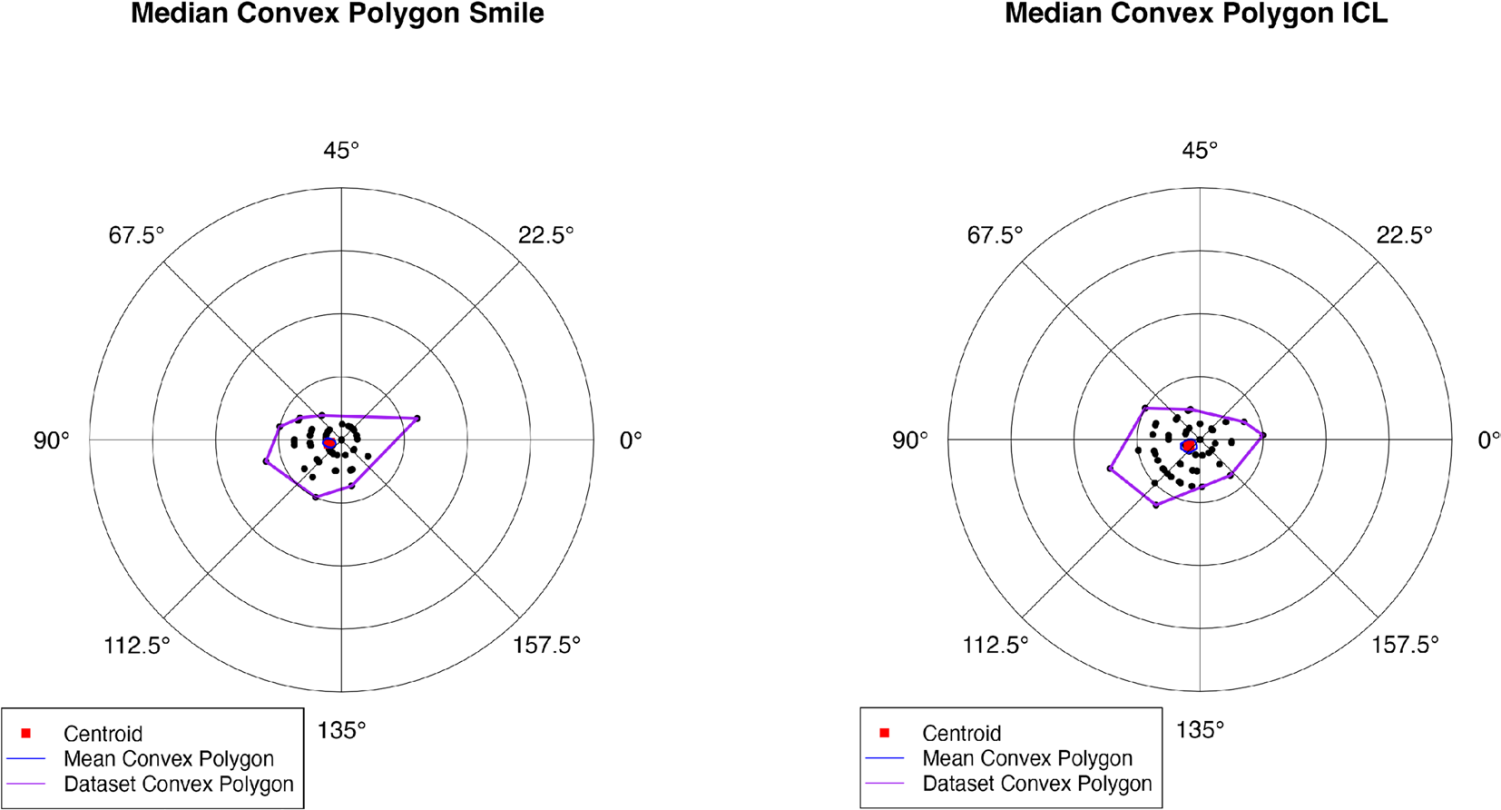
Polar plot of refractive cylinder magnitudes with median convex polygons for both groups. Each concentric ring corresponds to 1.0 diopter [8]

Both SMILE and toric ICL procedures demonstrated high efficacy, safety, and accuracy in correcting myopia with oblique astigmatism. The double-angle convex polygon plots Fig. 11. demonstrate that both groups exhibited well-centered centroids near the origin, indicating minimal systematic axis deviation. While outcomes were comparable, the ICL group showed slightly more predictable axis alignment, lower residual cylinder variance, and marginally superior UDVA performance. To confirm the adequacy of the sample size, a post hoc power analysis was performed for the correction index (CI), which demonstrated a moderate effect size (Cohen’s d = 0.48). With 70 eyes in each group and a significance level of α = 0.05, the achieved power was calculated to be 80.5%, indicating that the study was adequately powered to detect clinically relevant differences in astigmatic correction between SMILE and ICL.

### One year fellow-up

A 12-month follow-up analysis was performed for both groups to assess long-term visual and refractive stability.

In the ICL group, mean cylinder improved slightly from − 0.44 ± 0.35 D at 6 weeks (n = 70) to − 0.35 ± 0.25 D at 12 months (n = 28) (*p* = 0.044), indicating a small but statistically significant refinement of astigmatic correction. No significant changes were observed for spherical equivalent (+ 0.07 ± 0.44 D vs. − 0.05 ± 0.37 D; *p* = 0.976) or for sphere, UDVA, and CDVA (all *p* > 0.37). These findings confirm excellent refractive and visual stability after toric ICL implantation.

In the SMILE group, no significant differences were detected between 6 weeks (n = 70) and 12 months (n = 27) in cylinder (− 0.38 ± 0.29 D vs. − 0.38 ± 0.24 D; *p* = 0.412), sphere (*p* = 0.281), or spherical equivalent (− 0.16 ± 0.41 D vs. − 0.13 ± 0.36 D; *p* = 0.399). UDVA showed a mild, non-significant improvement (*p* = 0.160), consistent with gradual postoperative neural adaptation.

Collectively, these data demonstrate sustained refractive and visual stability in both SMILE and ICL groups over the first postoperative year, with minimal regression or drift.

## Discussion

Correction of oblique astigmatism poses a distinct clinical challenge due to the need for precise correction of both magnitude and axis [10–14]. Unlike the relatively straightforward correction of spherical myopia, oblique astigmatism is highly sensitive to axis misalignment, cyclotorsional movements, and biomechanical variations [11]. In this study, both Small Incision Lenticule Extraction (SMILE) and toric Implantable Collamer Lens (ICL) implantation demonstrated favorable safety and efficacy profiles for the correction of myopic oblique astigmatism. However, our findings suggest that toric ICL implantation may offer more predictable outcomes, even at moderate levels of preoperative astigmatism.

### Efficacy

At six weeks postoperatively, both groups showed excellent efficacy. The mean efficacy index was 0.97 ± 0.17 in the ICL group and 0.94 ± 0.15 in the SMILE group (*p* = 0.18). A postoperative uncorrected distance visual acuity (UDVA) equal to or better than preoperative corrected distance visual acuity (CDVA) was achieved in 83% of eyes in the ICL group and 79% in the SMILE group.

These findings underline the high efficacy of both methods for patients with oblique astigmatism.

Our findings are supported by a recent comparative analysis of FDA-reported outcomes, which evaluated SMILE, Toric ICL, and topography-guided LASIK (TG-LASIK) in the treatment of myopia and myopic astigmatism. In that study, all three modalities demonstrated high efficacy, with SMILE achieving significantly better accuracy for cylinder correction compared to Toric ICL across multiple thresholds (within ± 0.25 D, ± 0.50 D, and ± 1.00 D; all *p* < 0.01) [15]. These findings align with our results. Together, these data reinforce the suitability of both SMILE and ICL for effective visual rehabilitation in myopia, including eyes with oblique astigmatism.

These findings are in line with prior comparative studies of T-ICL, femto-LASIK, and SMILE in patients with myopic astigmatism. In a prospective study by Ganesh, all three modalities achieved effective astigmatism correction at one year, with mean residual cylinder values of –0.21 ± 0.28 D (T-ICL), − 0.17 ± 0.36 D (FS-LASIK), and − 0.22 ± 0.28 D (SMILE), showing no statistically significant differences in predictability (*p* > 0.05). In that study, 97% of SMILE-treated eyes and 93% of T-ICL eyes achieved UDVA of 20/20 or better, which aligns closely with our efficacy outcomes. Interestingly, the greatest gain in CDVA was observed in the T-ICL group, despite a small number of eyes requiring lens exchange due to rotation and vault-related issues. These results support the overall high efficacy of both SMILE and ICL, while also highlighting the importance of meticulous preoperative planning in phakic IOL implantation to minimize the risk of postoperative adjustments [16].

### Safety

The safety index was similarly high, with 1.02 ± 0.11 for ICL and 1.01 ± 0.09 for SMILE, and no eye in either group lost more than one line of CDVA. These findings align with numerous studies that have consistently reported excellent safety outcomes for both SMILE and toric ICL procedures [16–21].

### Accuracy

Regarding refractive accuracy, 85.7% of ICL eyes and 81.4% of SMILE eyes were within ± 0.50 D of the intended spherical equivalent. The mean achieved SEQ was 0.07 ± 0.44 D in the ICL group and − 0.16 ± 0.41 D in the SMILE group. Vector analysis revealed a mean correction index (CI) of 0.97 ± 0.32 in the ICL group and 1.04 ± 0.34 in the SMILE group (*P* = 0.48). The regression slope of surgically induced astigmatism (SIA) versus target-induced astigmatism (TIA) was 1.04 in the ICL group (R^2^ = 0.77) compared to 0.76 in the SMILE group (R^2^ = 0.67), indicating stronger vector predictability with ICL. Additionally, At one year postoperatively, both SMILE and toric ICL demonstrated excellent refractive stability, with no clinically significant regression or loss of visual acuity. The minor additional reduction in cylinder observed in the ICL group (mean ≈ 0.1 D, *p* = *0.044*) likely reflects subtle long-term axis stabilization rather than true regression.

the angle of error (AE) was lower in the ICL group, with 76% of eyes falling within ± 15° of the intended axis, compared to 82% in the SMILE group (*P* = 0.43). These results align with the findings of Ganesh et al., who reported comparable refractive predictability among T-ICL, femto-LASIK, and SMILE at one year [14, 16].

Our findings are further supported by a recent refraction-matched comparative study evaluating SMILE and ICL implantation for high myopia, which reported superior refractive predictability and visual quality in the ICL group. In that study, 90% of ICL-treated eyes achieved a spherical equivalent within ± 0.50 D of target, compared to 72.5% in the SMILE group (*p* = 0.045), confirming the higher precision of ICL in matching the intended refractive outcome. This aligns well with our own data. Additionally, the ICL group in that study showed significantly higher efficacy and safety indices, along with lower induction of higher order aberrations. Notably, patients in the ICL group reported fewer subjective visual disturbances such as starbursts and fluctuations of vision, supporting the qualitative advantage of ICL in visual performance. These findings emphasize that, while both procedures are highly effective, ICL may offer more consistent refractive accuracy and improved patient-reported visual quality, particularly in cases requiring high precision [20].

To confirm the adequacy of the sample size, a post hoc power analysis for CI was conducted. Based on the observed effect size (Cohen’s d = 0.48) and sample size of 70 eyes per group, the achieved power was 80.5%, indicating that the study was adequately powered to detect clinically relevant differences in astigmatic correction.

Postoperatively, SMILE produced outcomes in UDVA and SEQ comparable to FS-LASIK and transepithelial PRK (t-PRK), while demonstrating lower surgically induced astigmatism and coma index in other studies [22]. These advantages are attributed to its flapless approach and minimal epithelial remodeling, preserving corneal biomechanics and asphericity. However, despite these optical benefits, our data revealed higher residual cylinder and less predictable astigmatic correction with SMILE compared to ICL, likely due to the absence of real-time cyclotorsional alignment and reliance on manual centration.

A key limitation of the SMILE procedure is its lack of automated cyclotorsion compensation and intraoperative axis control. These limitations can result in axis misalignment during treatment, particularly in cases of oblique astigmatism, where small deviations can lead to significant undercorrection [23]. Recently, cyclotorsion tracking has been implemented in the ZEISS VISUMAX 800® platform. This system integrates OcuLign technology, which enables automatic compensation for cyclotorsion by detecting and correcting rotational misalignment intraoperatively. The addition of this feature represents a significant technological advancement, particularly for improving the accuracy of astigmatic correction in SMILE procedures. In patients with higher cylinder values, both procedures exhibited a tendency toward undercorrection, which was more pronounced in the SMILE group. This observation aligns with findings by Chow et al. and Ivarsen et al., who reported undercorrection rates of 13–16% per diopter in high astigmatism cases after SMILE.

In contrast, toric ICL implantation provides astigmatic correction independent of corneal shape and biomechanics. This may explain the more reliable axis alignment and lower residual cylinder observed in our ICL group. These findings are in line with previous studies by Shimizu et al. and Kamiya et al., who reported higher predictability and rotational stability with toric ICLs, especially in cases of high myopic astigmatism [24, 25]. One potential limitation of toric ICL treatment is the risk of postoperative lens rotation, which can lead to residual astigmatism and reduced visual quality [26]. Studies have shown that every 3° of rotational misalignment reduces the effectiveness of astigmatic correction by approximately 10%, with rotations greater than 10° significantly compromising visual outcomes [27, 28].

Undercorrection of astigmatism following SMILE surgery has been well-documented in the literature. Multiple factors may contribute to reduced astigmatic correction efficacy, including cyclotorsional misalignment, centration errors, and the magnitude of preoperative astigmatism. Researchers have proposed various strategies to address this issue [11, 13, 29–31]. No standardized nomogram protocol is widely accepted, leading to variability in refractive outcomes, particularly in cases of oblique or high astigmatism. Oblique astigmatism remains especially challenging, with no routinely adopted nomogram adjustments to date. In this context, our findings provide clinically meaningful data to support refinement of nomogram strategies for oblique astigmatism, where precise alignment and magnitude correction are crucial to achieving optimal outcomes. Recently Yu et al. developed and validated a regression-based nomogram for the correction of high myopic astigmatism using SMILE surgery. Their method was based on a linear regression between target-induced astigmatism (TIA) and flattening effect (FE), applied to 112 eyes with astigmatism ranging from − 2.75 to − 4.50 D. Compared to a control group of 143 eyes treated using manifest refraction alone, the nomogram group showed significantly higher accuracy, with 97% of eyes within ± 0.50 D of residual astigmatism versus 89% in the control group (*P* = 0.010). The angle of error was also improved, with 100% of eyes in the nomogram group within ± 5°, compared to 95.8% in the control group (*P* = 0.028). Vector analysis demonstrated reduced undercorrection in the nomogram group (mean 2.7% overcorrection) compared to the manifest group (mean 8.4% undercorrection). These findings support the clinical utility of regression-based nomogram refinement for enhancing predictability in high astigmatism SMILE treatments [32].

To mitigate cyclotorsional effects in SMILE, methods like limbal marking and head positioning have been suggested, but these are manual and lack consistency. In contrast, toric ICLs allow for precise preoperative planning and offer greater rotational stability, reducing susceptibility to misalignment.

Our findings reflect real-world conditions and reaffirm the need for more standardized and robust alignment protocols in SMILE. The small supported differences in refractive predictability, particularly in high or oblique astigmatism, favor toric ICL as the more reproducible approach under current clinical practice.

At one year postoperatively, both SMILE and toric ICL demonstrated excellent refractive stability, with no clinically significant regression or loss of visual acuity. The minor additional reduction in cylinder observed in the ICL group (mean ≈ 0.1 D, *p* = *0.044*) likely reflects subtle long-term axis stabilization rather than true regression.

## Conclusion

Both SMILE and toric ICL implantation demonstrated good efficacy, safety, and predictability in the correction of myopic oblique astigmatism. Vector analysis revealed slightly more consistent axis alignment, and a closer match between intended and achieved astigmatic outcomes in the toric ICL group.

It should be noted, however, that toric ICLs are available only in 0.50 D cylinder increments, which may limit refractive fine-tuning in certain cases. SMILE offers notable advantages in terms of corneal preservation and biomechanical integrity, but its lack of intraoperative cyclotorsion control remains a limitation—particularly for eyes with oblique astigmatism. Ongoing software advancements may help address this limitation, and further studies are warranted to assess their clinical impact.

Our findings support the refinement of nomograms for oblique astigmatism correction, particularly in SMILE, where rotational misalignment remains a key variable. Toric ICL implantation benefits from digital surgical planning and intraoperative axis control, which may help reduce variability, even in less experienced hands. In contrast, SMILE remains more operator-dependent, with a steeper learning curve in managing high or oblique cylinder cases.

A tailored approach may therefore be appropriate: SMILE could be favored in patients with low to moderate astigmatism and stable corneal architecture, whereas toric ICL may be preferred in cases of high or oblique astigmatism where rotational precision is critical.

From a clinical perspective, toric ICL may be preferable in eyes with higher or more irregular oblique astigmatism, while SMILE remains an effective option for cases with lower to moderate astigmatic magnitudes. These findings highlight the importance of tailoring the surgical approach to the severity and orientation of astigmatism rather than applying a uniform treatment strategy.

### Limitation

This study has several limitations that should be acknowledged. Its retrospective design may restrict the generalizability of the findings. Although the inclusion of one-year follow-up data strengthens the assessment of long-term stability, a substantial loss to follow-up occurred, as not all patients returned for the 12-month examination. This was partly related to the referral nature of the cohort, with many patients continuing postoperative care with local ophthalmologists. Furthermore, longer observation periods beyond one year are necessary to evaluate the durability of outcomes and possible late changes.

In addition, subgroup analyses based on corneal thickness and gender were not feasible due to the limited sample size after strict matching for refractive parameters. Larger, prospectively designed studies are needed to address these factors and to confirm whether they influence surgical outcomes.

Future prospective, multicenter studies with extended follow-up would provide a more comprehensive understanding of long-term efficacy and stability in eyes with oblique astigmatism.

**Prior presentation**: Parts of this study were presented at the American Academy of Ophthalmology Annual Meeting (AAO), 2025.

## Data Availability

The authors confirm that the data supporting the findings of this study are available within the article and its supplementary materials.
